# Genomic and evolutionary characteristics of G9P[8], the dominant group a rotavirus in China (2016–2018)

**DOI:** 10.3389/fmicb.2022.997957

**Published:** 2022-09-16

**Authors:** Xiafei Liu, Mengxuan Wang, Shan Li, Jingxin Li, Jinbo Xiao, Huiying Li, Qing Zhang, Xiangyu Kong, Hong Wang, Dandi Li, Zhaojun Duan

**Affiliations:** ^1^Chinese Center for Disease Control and Prevention, National Institute for Viral Diseases Control and Prevention, Beijing, China; ^2^School of Public Health, Gansu University of Chinese Medicine, Lanzhou, China

**Keywords:** G9P[8], rotavirus, whole-genome, Bayesian analysis, China

## Abstract

G9P[8] became the predominant rotavirus A (RVA) genotype in China in 2012. To evaluate its genetic composition at the whole-genome level, 115 G9P[8] RVA strains isolated from children under 5 years old were sequenced and characterized. All 13 strains in 2016 and 2017 and an additional 54 strains in 2018 were genotyped as G9-P[8]-I1-R1-C1-M1-A1-N1-T1-**E1**-H1. The other 48 strains in 2018 were all genotyped as G9-P[8]-I1-R1-C1-M1-A1-N1-T1-**E2**-H1, with the NSP4 gene characterized as a DS-1-like genotype. The time of the most recent common ancestor (tMRCA) and evolution rates of the VP7, VP4, and NSP4 (E1 and E2) genes of these strains were estimated by Bayesian evolutionary dynamics analysis. We estimated the evolution rates (nt substitutions per site per year) as 1.38 × 10^–3^ [the 95% highest posterior density (HPD) was 1.09–1.72 × 10^–3^] for VP7, 0.87 × 10^–3^ (95% HPD: 0.75–1.00 × 10^–3^) for VP4, 0.56 × 10^–3^ (95% HPD: 0.41–0.73 × 10^–3^) for NSP4-E1, and 1.35 × 10^–3^ (95% HPD: 0.92–1.86 × 10^–3^) for NSP4-E2. The tMRCA was estimated to be 1935.4 (95% HPD: 1892.4–1961.3) for VP7, 1894.3 (95% HPD: 1850.5–1937.8) for VP4, 1929.4 (95% HPD: 1892.4–1961.3) for NSP4-E1, and 1969.2 (95% HPD: 1942.2–1985.3) for NSP4-E2. The baseline genetic information in this study is expected to improve our understanding of the genomic and evolutionary characteristics of the rotavirus genome. Furthermore, it will provide a basis for the development of next-generation rotavirus vaccines for humans.

## Introduction

Human rotavirus group A (RVA) is a leading cause of acute gastroenteritis (AGE) in infants worldwide (Parashar et al., 2003). Although vaccination against rotaviruses has reduced hospitalization and mortality rates, there were still more than 128,500 deaths and 258,000,000 episodes of diarrhea among children under 5 years old due to RVA throughout the world in 2016 (Troeger et al., 2018). RVA is a member of the Reoviridae family and has an 11-segment dsRNA genome that encodes six structural proteins (VP1–4, VP6, and VP7) and six non-structural proteins (NSP1–NSP5/6) (Estes and Cohen, 1989). Based on the phylogenies of the capsid protein VP7 and spike protein VP4, at least 41 G genotypes and 57 P genotypes have been identified in human and animal RVA strains.^1^ Of these, G9P[8] has become the fifth dominant genotype after G1P[8], G2P[4], G3P[8], and G4P[8], and has spread globally over the past two decades (Rahman et al., 2005; Matthijnssens et al., 2010). RVA G9P[8] was first isolated from a child with gastroenteritis in the United States and named WI61 in 1983 (Clark et al., 1987). Since then, it has spread quickly worldwide and was subsequently reported in Asia (Chan-It and Chanta, 2018), Europe (Pothier, 2010; Kaplon et al., 2018), and Africa (Ouermi et al., 2017). In mainland China, G9P[8] became the predominant RVA genotype in 2012 (Tian et al., 2018; Yu et al., 2019; Kuang et al., 2020), and subsequently accounted for 74.05, 77.44, and 87.58% of RVA-positive samples in 2016, 2017, and 2018, respectively (undisclosed data from the National Surveillance Report of Viral Diarrhea in China, 2016–2018).

Based on the genotype constellations of VP7-VP4-VP6-VP1-VP2-VP3-NSP1-NSP2-NSP3-NSP4-NSP5/6 genes, human RVA has been mostly classified into three genogroups: Wa-like (Gx-P[8]-I1-R1-C1-M1-A1-N1-T1-E1-H1), DS-1 like strains (GxP[4]-I2-R2-C2-M2-A2-N2-T2-E2-H2), and AU-1-like strains (G3-P[9]-I3-R3-C3-M3-A3-N3-T3-E3-H3) (Matthijnssens et al., 2008a,b, 2011). The G1, G3, G4, G9, and G12 viruses are usually clustered into the Wa-like constellation, and the G2 and G8 viruses are usually clustered into the DS-1-like constellation (Heiman et al., 2008). Using this classification, multiple genotypes of rotavirus have been assigned, and their phylogenetic relationships can be analyzed further. To obtain conclusive data on the overall genetic makeup and evolutionary patterns of common RVAs, whole-genome analysis of dominant RVA strains detected on a large scale is essential (Ghosh and Kobayashi, 2011). G9P[8] RVA has been the predominant genotype since 2012 in China (Zhou et al., 2020). Whole-genome phylogenetic analysis may reveal the genetic evolution, reassortment characteristics, and genetic mechanisms underlying its successful spread.

The World Health Organization (WHO) recommends the use of two live vaccines, Rotarix and RotaTeq, for the prevention of severe symptoms due to rotavirus infection in children. However, only the RotaTeq and Lanzhou lamb rotavirus vaccine (G10P[15]) are commonly used in China and the vaccine coverage was only about 20% (Bergman et al., 2021). The rotavirus virion is composed of two capsid proteins, VP4 and VP7, which contain the major neutralizing antigen epitopes. Antigenic and genetic characterization of dominant wildtype rotavirus strains are essential for the prediction and evaluation of the potential efficacy of any rotavirus vaccine, and are also useful for the development or improvement of vaccines. A DS-1-like NSP4 gene from Wa-like G9P[8] backbone strains has been found in China (Lu et al., 2020) and Japan (Fujii et al., 2020). Considering the predominance of the G9P[8] rotavirus in China in the last decade, we performed a comprehensive analysis of the complete genomes of 115 G9P[8] RVA strains, and analyzed the evolutionary relationships and rates of evolution of the VP7, VP4, and NSP4 genes in this study to provide more information on the evolution of RVAs and the development of next-generation rotavirus vaccines for use in humans.

## Materials and methods

### Sample collection

**FIGURE 1:**
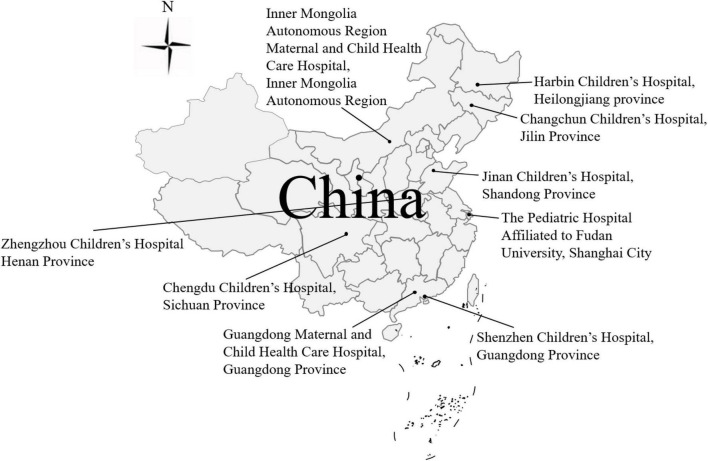
The 115 dominant G9P[8] RVA strains collected from eight sentinel hospitals in seven provinces in China from 2016 to 2018.

Surveillance was conducted at sentinel hospitals in different provinces in China. Children under 5 years who were admitted to the hospital with three or more passages of watery diarrhea or looser-than-normal stool in less than 24 h were considered to have gastroenteritis. Samples were obtained with oral consent from the parents/guardians of the patients. A total of 3819 RVA-positive stool samples were collected and genotyped during the three seasons from 2016 to 2018; 79.69% were positive for G9P[8] (data from the National Surveillance Report of Viral Diarrhea in China, 2016–2018). A total of 115 G9P[8] stool samples collected from nine sentinel hospitals (Harbin Children’s Hospital, Changchun Children’s Hospital, Inner Mongolia Autonomous Region Maternal and Child Health Care Hospital, Jinan Children’s Hospital, Zhengzhou Children’s Hospital, Chengdu Children’s Hospital, Guangdong Maternal and Child Health Care Hospital, Shenzhen Children’s Hospital, and the Pediatric Hospital Affiliated to Fudan University) (Figure 1 and Supplementary Table 1) were used for whole-genome sequencing.

### Whole-genome sequencing

The RNA genomes of the 115 stool samples were extracted using a QIAamp Viral RNA Mini Kit (Qiagen, Germantown, MD, United States), then amplified using previously published primer sets (Matthijnssens et al., 2008a) and a One Step RT-PCR Kit (Qiagen), in accordance with the manufacturer’s instructions. The PCR products were sent to Tsingke Biotechnology Co., Ltd. (Beijing, China) for sequencing. Nucleotide sequences were determined using an ABIPRISM 3730 automated DNA sequencer (Thermo Fisher). Whole genome sequences excluding primer sequences were obtained through adapter trimming and assembly using SeqMan software (DNAStar 5.0). After sequencing and assembly, nearly full-length sequences (except for the 5′- and 3′-terminal sequences) were obtained. The genotype of each genome segment was preliminarily determined using the Rotavirus A Genotype Determination online genotyping tool.^2^

### Phylogenetic analysis

A total of 1,265 near-full-length gene sequences from the 115 strains were aligned with reference sequences using CLUSTAL W in the MEGA software package, v.7.0.21 (Wang et al., 2014). The nucleotide and amino acid identities were calculated used BioEdit 7.1.3.0. Genetic distances were calculated with the Tamura three-parameter method at the nucleotide level, and phylogenetic trees were constructed in MEGA v.7.0.21 based on the maximum likelihood (ML) method with 1000 bootstrap replicates for branch support. Reference sequences were retrieved from GenBank and the lineage designations were defined based on previous studies for VP7 (Phan et al., 2007), VP4 (Espinola et al., 2008), and NSP4 trees (Fujii et al., 2020; Lu et al., 2020).

### Bayesian evolutionary dynamics analysis

A Bayesian time-scaled tree of the VP7, VP4, and NSP4 genes was generated for further evolutionary analysis with our 115 strains and other representative strains, retrieved from GenBank (208 VP7 references, 134 VP4 references, 96 NSP4-E1 references, and 100 NSP4-E2 references, Supplementary Tables 2–5) in BEAST v.1.8.4 (Drummond et al., 2012). Ambiguous strains with an unknown collection year and strains associated with vaccines, cultivated in eukaryotic cells, from environmental samples, and presenting recombination signals as determined using the Recombination Detection Program (RDP) v.4.56 were also excluded (Martin et al., 2015). Based on the TempEst v1.5.3, root-to-tip regression analysis was performed to ensure the dataset had a temporal signal and all strains confirmed to a linear rate of evolution (Rambaut et al., 2016). The TIM + G (VP7 and VP4 genes) and HKY + G (NSP4-E1 and -E2 genes) nucleotide substitution models, an uncorrelated lognormal relaxed clock model (Drummond et al., 2006), and a coalescent constant-size tree prior (Drummond et al., 2005) were utilized. Convergence of the Markov chain Monte Carlo sample on the posterior distribution was defined at an effective sample size > 200 when 100 million MCMC runs with sampling every 1000 steps were carried out, as calculated after a 10% burn-in with Tracer v.1.7.1.^3^ The maximum clade credibility (MCC) tree was annotated using TreeAnnotator v.1.8.4 and visualized in FigTree v.1.4.4.^4^

## Results

### Full-genome sequencing

The genotype constellations of all 115 G9P[8] RVA samples collected from 2016 to 2018 were analyzed, and 11 strains in 2016, 2 strains in 2017, and 54 strains in 2018 were genotyped as G9-P[8]-I1-R1-C1-M1-A1-N1-T1-E1-H1, which is closely related to the Wa-like genotype 1 constellation. However, the other 48 strains in 2018 were genotyped as G9-P[8]-I1-R1-C1-M1-A1-N1-T1-E2-H1, with the NSP4 gene characterized as a DS-1 like genotype.

All obtained genomic sequences were deposited in GenBank (ON991952–ON993216).

### Phylogenetic analysis

**FIGURE 2:**
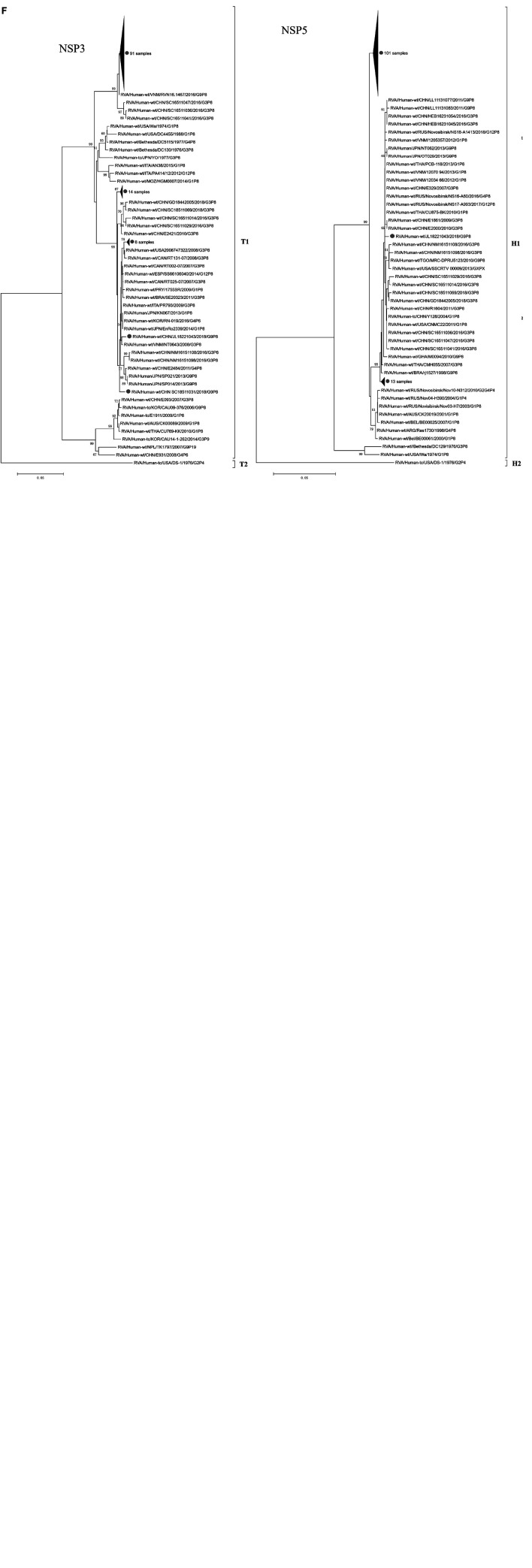
Phylogenetic dendrograms based on the nucleotide sequences of the 115 gene segments of rotaviruses for VP7 **(A)**; VP4 **(B)**; NSP4 **(C)**; VP1-VP3 **(D)**; VP6, NSP1, and NSP2 **(E)**; NSP3 and NSP5 **(F)** in China from 2016 to 2018 (•). In VP4 vaccine strains are reported in bold. Trees were constructed by the maximum-likelihood method with 1,000 bootstrap replicates.

In the VP7 phylogenetic tree (Figure 2A), all G9 strains could be divided into six distinct lineages: lineages I and II contained the first described G9 strains WI61 and F45 from the United States, AU32 from Japan, and 116E from India isolated in the 1980s; lineages IV and V were isolated in the 1990s, with the typical strains being 97’sz37 exclusively in China, and OM46 and om67 exclusively in the United States. Lineage VI, which was first detected in 1997–2002, included strains mainly distributed in Asia, especially in China and Japan. In our study, all of the strains were clustered into lineage VI (98.4–100% nt and 98.4–100% aa similarity), except strain SZ18442196, which segregated in lineage III. Indeed, lineage III, which emerged/reemerged in the mid-1990s, was more widely distributed than lineage VI and accounted for the most prevalent G9 viruses throughout the world.

In the VP4 phylogenetic tree (Figure 2B), P[8] strains could be divided into four distinct lineages: two rotavirus vaccines Rotarix™ (G1P[8]; GlaxoSmithKline Biologicals, Wavre, Belgium) and RotaTeq™ (G1-G4P[8]; Merck & Co., Inc., Whitehouse Station, NJ, United States) were clustered in lineages II and I, respectively. Lineage III is the most prevalent lineage globally at present. In our study, with the exception of SD18370120, which segregated in lineage IV, all of the remaining 114 strains were clustered into lineage III, which had nucleotide and amino acid identities ranging from 96.1 to 100% and 97.5 to 100%, respectively. Lineage IV (MW670-like P[8]) is a rare P[8] subtype in China, which emerged in 2008 (Z1108) and 2009 (E1545) in Wuhan, and subsequently in 2012 (JS2012) in Jiangsu. These four G9P[8] strains from China in lineage IV had 98.4–99.5% and 98.9–99.3% sequence identities at the nucleotide and amino acid levels, respectively, and clustered with the worldwide strains G1P[8], G2P[8], G3P[8], and G4P[8].

A phylogenetic analysis based on the NSP4 sequences showed that 67 strains belonged to the E1 cluster, with nucleotide and amino acid identities of 95.8–100% and 96.5–100%, respectively, and 48 strains belonged to the E2 cluster, with nucleotide and amino acid identities of 97.4–100% and 98.2–100%, respectively (Figure 2C). Furthermore, the E1 cluster was divided into three lineages: the rotavirus vaccine strain Rotarix™ (G1P[8]; GlaxoSmithKline Biologicals) and strain Wa G1P[8] were clustered in lineage I. All the strains in our study were clustered into lineage III, except SC18511031 and HEB16231043, which belonged to lineage II. The E2 cluster was further divided into four lineages: the 48 E2 strains in our study were all clustered into lineage IV, which was distinct from the rotavirus vaccine strain RotaTeq™ (G1–G4P[8]; Merck & Co., Inc.) in lineage I, G3P[8] strains SKT-281 and D388 in lineage II, and G2P[4] strain DS-1 in lineage III. The 47/48 E2 strains in lineage IV were highly similar to the G2P[4] strains MU14-16, Tokyo 17-15, and SP012 from Japan and the G9P[8] strains E6398, L2448, and Z2768 from China; the other strain, GD18442004, showed a sister group relationship with G2P[4] strains DPRU295 and DPRU296 from Mauritius.

### Bayesian evolutionary dynamics analysis

The tMRCA was estimated to be 1935.4 (95% HPD: 1892.4–1961.3) for VP7 strains, 1894.3 (1850.5–1937.8) for VP4 strains, 1929.4 (1892.4–1961.3) for NSP4-E1 strains, and 1969.2 (1942.2–1985.3) for NSP4-E2 strains. The estimated evolutionary rates were 1.38 × 10^–3^ nucleotide substitutions per site per year (95% highest posterior density [HPD] 1.09–1.72 × 10^–3^) for VP7 strains, 0.87 × 10^–3^ (0.75–1.00 × 10^–3^) for VP4 strains, 0.56 × 10^–3^ (0.41–0.73 × 10^–3^) for NSP4-E1 strains, and 1.35 × 10^–3^ (0.92–1.86 × 10^–3^) for NSP4-E2 strains (Table 1).

**TABLE 1:** Evolutionary rates of genes and tMRCA of lineages determined by Bayesian phylogenetic reconstruction using MCMC analysis in BEAST for the VP7, VP4, and NSP4 genes.

Parameter	Genome segment			
	
	VP7	VP4	NSP4-E1	NSP4-E2
Number of sequences	323	249	163	148
Sampling interval	1986–2019	1976–2020	2004–2019	1987–2019
Sampling coverage	Worldwide	Worldwide	Worldwide	Worldwide
Evolutionary rate (× 10^–3^) (95% HPD interval)	1.38 (1.09–1.72)	0.87 (0.75–1.00)	0.56 (0.41–0.73)	1.35 (0.92–1.86)
tMRCA (95% HPD interval)	1935.4 (1892.4–1961.3)	1894.3 (1850.5–1937.8)	1929.4 (1892.4–1961.3)	1969.2 (1942.2–1985.3)

**FIGURE 3:**
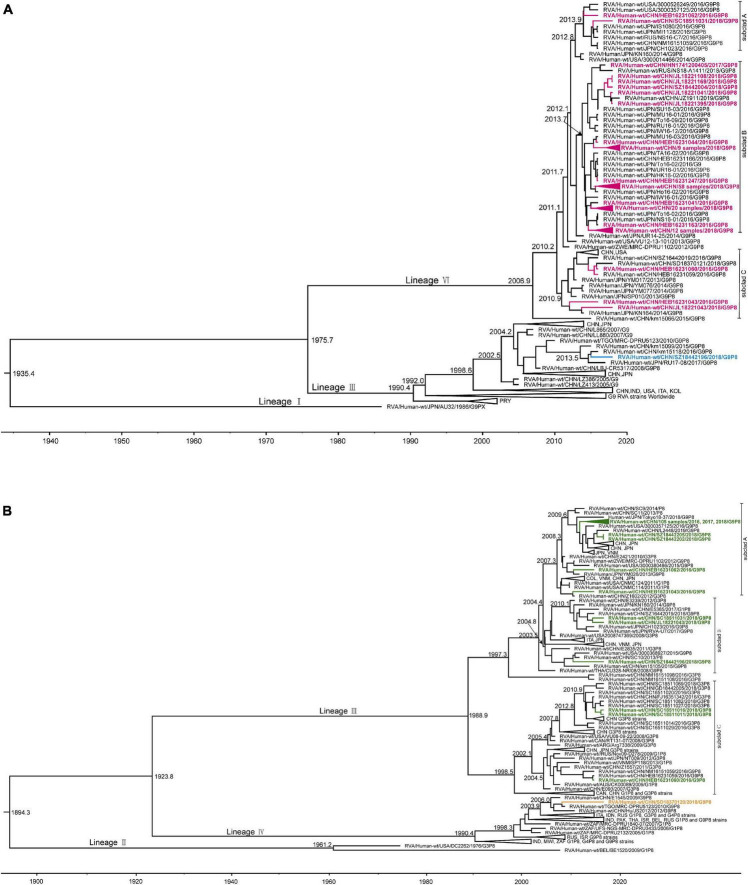
Maximum clade credibility (MCC) tree of VP7 **(A)** and VP4 **(B)** for G9P[8] rotavirus with collapsed branches corresponding to the lineage subclades. The MCC tree was constructed by Bayesian Markov chain Monte Carlo analysis implemented in BEAST v. 1.8.4. Reference strain sequences were from GenBank (http://www.ncbi.nlm.nih.gov). The countries of isolation (ISO 3166-1-alpha-3 codes) and sampling times of the strains are indicated. In the VP7 MCC tree **(A)**, the tree branches for study strains of lineage VI are amaranth and those for lineage III are blue. In the VP4 MCC tree **(B)**, the tree branches for study strains of lineage III are green and those for lineage IV are orange.

For the VP7 MCC tree (Figure 3A), as representative strains of lineages II, IV, and V were cultured in eukaryotic cells and lineage I was the early G9 strain AU32 collected from Japan in 1986, lineage III and lineage VI were selected for further analysis. Lineage III, which included the majority of the currently circulating G9P[4], G9P[6], and G9P[8] strains worldwide, was traced back to 1990.4 with a 95% HPD interval of 1984.0–1995.1. In our study, only one strain, SZ18442196, which was collected from Shenzhen, China, in 2018, was clustered into lineage III and showed a very close evolutionary relationship with strains km15099 and km15118 collected from Kunming, China, in 2015 and 2016, and strain RU17-08 collected from Japan in 2017. The tMRCA of the four strains was calculated to be 2013.4 (2011.9–2014.7). Compared with lineage III, lineage VI was younger, and was traced back to 2007.0 with a 95% HPD interval of 2001.7–2010.0; the other 114 strains in our study were all clustered into this lineage. For descriptive purposes, the lineage VI strains were further divided into three subclusters (designated A, B, and C). The two studied strains collected from Harbin and Chengdu in subcluster A showed a very close evolutionary relationship with strains from the United States, Japan, and Russia, such as 3000357125, CH1023, and NS16-C7, and the tMRCA was 2012.8 (2011.9–2013.6). The 113 studied strains collected from Harbin, Changchun, Hohhot, Jinan, Zhengzhou, Chengdu, Guangzhou, Shenzhen, and Shanghai in subcluster B showed a very close evolutionary relationship with strains from China, Japan, and Russia, such as JZ1911, U16-03, and NS18-A1411, and the tMRCA was 2012.8 (2011.9–2013.6). The three studied strains collected from Harbin and Changchun in subcluster C showed a sister relationship with strains from Japan, such as YM017 and KN164, and the tMRCA was 2010.9 (2009.6–2012.2).

For the VP4 MCC tree (Figure 3B), as the representative strains of lineage I were cultured in eukaryotic cells and lineage II consisted of strains DC2262 collected from the United States in 1976 and BE1520 collected from Belgium in 2009, lineage III and lineage IV were selected for further analysis. Lineage IV, which contained the majority of the currently circulating G1P[8], G4P[8], and G9P[8] strains worldwide, was traced back to 1990.4 with a 95% HPD interval of 1985.6–1993.2. In our study, only one strain, SD18370120, collected from Jinan in 2018, was clustered into lineage IV and was clustered along with strain MRC-DPRU5123 collected from Togo in 2010, which showed a sister relationship with strains E1545 from Wuhan, China, in 2009 and JS2012 from Suzhou, China, in 2012. The tMRAC of these four strains was 2006.0 with a 95% HPD interval of 2004.4–2007.2. Compared with lineage IV, lineage III was older, and was traced back to 1988.9 with a 95% HPD interval of 1980.7–1995.0. Similar to the VP7 MCC tree, the other 114 strains in our study were all clustered into lineage III, which is currently the most prevalent lineage globally. For descriptive purposes, the lineage III strains were further divided into three subclusters (designated A, B, and C). A total of 109 of 114 strains collected from Harbin, Changchun, Hohhot, Jinan, Zhengzhou, Chengdu, Guangzhou, Shenzhen, and Shanghai were divided into subcluster A, which was traced back to 2007.3 with a 95% HPD interval of 2006.1–2008.2, and showed a very close evolutionary relationship with strains from China, Japan, and the United States, such as L2448, Tokyo18-37, and 3000357125. The three studied strains collected from Changchun, Chengdu, and Shenzhen in subcluster B showed a very close evolutionary relationship with strains from China and Japan, such as SC10 and CH1023, and the tMRCA was 2003.6 (2001.6–2005.4). The three strains collected from Harbin and Chengdu in subcluster C were almost exclusively of the G3P[8] genotype, with the exception of three G9P[8] strains in our study, which clustered along with G3P[8] strains from China, and the tMRCA was 1998.5 (1994.4–2002.0).

## Discussion

It has been suggested that rotavirus evolution occurs mainly through the selection of point mutations or reassortment of the segmented genome (McDonald et al., 2009). Across the VP1–VP3, VP6, NSP1–NSP3, and NSP5 trees, the 115 strains exhibited the same pattern (Figures 2D–F); they clustered with the same group of contemporary Chinese strains and were members of conserved clades consisting of G3/G1/G9P[8] R1-C1-I1-M1-A1-N1-T1-E1-H1 strains, similar to the findings for VP7 and VP4 phylogenetic trees. However, not all segments from the same strain always clustered together, suggesting that interstrain reassortment may occur in some G9P[8] strains.

Genome segment exchanges have occasionally been reported in many different genotypes, and even if they did emerge, the reassorted viruses may be less fit for the environment than their parental strains and unable to compete with them (Yamamoto et al., 2014). Therefore, human rotaviruses with a Wa-like or a DS-1-like genome constellation were more common. However, the 48/115 Wa-like G9P[8] strains with a DS-1-like segment of NSP4 detected here show that this reassorted genotype is common and genetically stable. This reassorted genotype was first reported in Jinzhou, China (Lu et al., 2020), and Tokyo, Japan (Fujii et al., 2020) in 2018, and our results indicate the wide circulation of G9-P[8]-I1-R1-C1-M1-A1-N1-T1-E2-H1 strains in China in 2018. A previous study showed that the G9P[8]-E2 strains were generated by intergenogroup reassortment between commonly circulating G9P[8] and G2P[4] strains, and no characteristic substitutions and no obvious changes in virulence were observed in the G9P[8]-E2 strains [18–19], but it was difficult to determine the origins of these strains.

A previous study suggested that reassortment seems to occur more frequently in the NSP4 segment of G1P[8] RVA than other segments (Fujii et al., 2019), and its evolutionary rate of 1.40 × 10^–3^ was higher from 1987 to 2000 [31] and 1.01 × 10^–3^ from 1999 to 2011 (Tatsumi et al., 2014; Zeller et al., 2015). However, in our study (Table 1), the evolutionary rate of G9P[8]-E1 strains from 1980 to 2019 was markedly lower with a value of 0.56 × 10^–3^ (0.41–0.73 × 10^–3^). In contrast, the evolutionary rate of G9P[8]-E2 strains from 1987 to 2019 was 1.35 × 10^–3^ (0.92–1.86 × 10^–3^), which was obviously higher than for G9P[8]-E1. As the exceptional tendency for substitutions in NSP4 may affect the frequency of reassortments, further study is needed to determine whether this reassortment event was associated with the rapid evolution of G9P[8]-E2. Obtaining sequence information from wild-type clinical strains may be helpful for developing vaccination strategies. Therefore, continuous investigation and the accumulation of additional samples based on full-genome analysis, including unusual reassortants, are necessary to obtain more comprehensive epidemiological data for RVA.

Next, a time-scaled Bayesian phylogenetic tree was constructed to examine the evolution of G9P[8] RVAs (Figures 3A,B). The tMRCA of G9 was estimated to be 1935.4 (1892.4–1961.3) and the evolutionary rate of G9 was estimated to be 1.38 × 10^–3^ (1.09–1.72 × 10^–3^) nucleotide substitutions per site per year, similar to previous reports (Matthijnssens et al., 2010; Dian et al., 2017; Fujii et al., 2019). Comparisons with previous reports indicated that the VP7 segment in G9 strains had a faster evolutionary rate than those in G1 (5.99–8.63 × 10^–4^), G2 (5.66–9.196 × 10^–4^), and G3 (7.34 × 10^–4^) strains (Fujii et al., 2019). An analysis of the tMRCA of prevalent RVA strains enabled predictions of their rate of spread; 3–5 years would pass from the generation of an ancestor virus to the epidemic spread of that virus throughout China. The evolutionary rate of P[8] was estimated to be 0.87 × 10^–3^ (0.75–1.00 × 10^–3^) nucleotide substitutions per site per year, similar to a previous report for G1P[8] (Tatsumi et al., 2014), but slower than for the G9 segment. As the viral prevalence and evolutionary rates may change with the widespread use of vaccines worldwide, it is important to calculate the evolutionary rate of prevalent RVAs to deduce the epidemics of the genotype constellation.

The genotype shift and changes in evolutionary rates between before and after the introduction of vaccines were also reported in several countries (Mukhopadhya et al., 2017; Lartey et al., 2018; Carvalho-Costa et al., 2019; Hungerford et al., 2019). At present, two oral vaccines, Rotarix™ and RotaTeq™, are in use worldwide; however, neither includes the G9 genotype. It has been suggested that the genotypes of RVA strains with different antigenic properties can enable rotaviruses to escape adaptive immunity (Zeller et al., 2012). To date, no significant evidence of a vaccine-attributable increase in prevalence of G9 has been reported. However, there is concern about selective pressure on prevalent rotavirus strains and possible changes in their evolutionary rates, which may lead to the spread of new rotavirus genotype or subgenotypic lineages worldwide [5]. In the present study, the G9 lineage of the most prevalent strains (lineage IV) in China and Japan was distant from the worldwide strains (lineages III); further investigation is required to determine the reasons for this trend. The VP4 phylogenetic tree showed that the most prevalent P[8] lineage strains in this study (lineage III) were evolutionarily distant from the vaccine strains (lineages I and II). In addition, one strain, SD18370120, isolated from Jinan in 2018, belonged to the rare P[8] subtype (lineage IV) of the VP4 gene (MW670-like P[8]), which was first identified in Wuhan, China, in 2008 and 2009 (Wang et al., 2011), and subsequently reported sporadically in Jiangsu, China (Xu et al., 2018). Therefore, continuing analysis of the genomic characteristics of G9P[8] RVA is warranted to monitor the changes in antigen-specific epitopes of these prevalent strains.

In conclusion, this large-scale whole genome-based study was performed to assess the dominant G9P[8] RVA in an Asian country. The genetic information in this study represents baseline data to improve our understanding of the genomic and evolutionary characteristics of the rotavirus genome, which will be helpful for the evaluation and development of rotavirus vaccines for humans.

## Data availability statement

The data presented in this study are deposited in the Genbank repository, accession number: ON991952–on993216.

## Ethics statement

This study was reviewed and approved by the National Institute for Viral Diseases Control and Prevention (Beijing, China). The guardians of the recruited children in this study were informed of the aims of this investigation and provided oral consent.

## Author contributions

XL, DL, and ZD conceived and designed the study. MW and SL performed RNA extraction, sequencing, and sequences classification. JL performed molecular analyses. XL and JX contributed to phylogenetic analyses and Bayesian evolutionary analysis. HL, QZ, XK, and HW participated in sample collection and data analysis. XL wrote the manuscript. ZD and DL were responsible for the critical revision of the manuscript. All authors have received the final version of the manuscript.

## Footnotes

1
https://rega.kuleuven.be/cev/viralmetagenomics/virus-classification/rcwg

2
https://www.viprbrc.org/brc/rvaGenotyper.spg?method=ShowCleanInputPage&decorator=reo

3
http://tree.bio.ed.ac.uk/software/tracer

4
http://tree.bio.ed.ac.uk/software/figtree/
