# Supplementary material for: Genomic and evolutionary characteristics of G9P[8], the dominant group a rotavirus in China (2016–2018)
Source: Front Microbiol. 2022 Sep 16;13:997957. doi: 10.3389/fmicb.2022.997957 (PMC9522900; doi:10.3389/fmicb.2022.997957)
Supplement: Supplementary file 1 [file Table_1.DOCX]

**Supplementary material.**

**Table S1** The information about the 115 dominant G9P[8] RVA strains collected from nine sentinel hospitals in eight provinces in China from 2016 to 2018.

| Location | Sentinel hospitals | No. of Strains | | | |
| --- | --- | --- | --- | --- | --- |
|  |  | **2016**  **(NSP4-E1)** | **2017**  **(NSP4-E1)** | **2018** | |
|  |  |  |  | **NSP4-E1** | **NSP4-E2** |
| Heilongjiang | Harbin Children’s Hospital | 11 | 0 | 0 | 0 |
| Jilin | Changchun Children’s Hospital, | 0 | 0 | 19 | 7 |
| Inner Mongolia | Inner Mongolia Autonomous Region Maternal and Child Health Care Hospital | 0 | 1 | 0 | 0 |
| Shandong | Jinan Children’s Hospital | 0 | 0 | 2 | 1 |
| Henan | Zhengzhou Children’s Hospital | 0 | 1 | 0 | 0 |
| Sichuan | Chengdu Children’s Hospital | 0 | 0 | 10 | 6 |
| Shanghai | The Pediatric Hospital Affiliated to Fudan University | 0 | 0 | 0 | 4 |
| Guangdong | Guangdong Maternal and Child Health Care Hospital | 0 | 0 | 9 | 5 |
|  | Shenzhen Children’s Hospital | 0 | 0 | 14 | 25 |

**Table S2** The 208 VP7 strains used for evolutionary analyses in the MCC tree of G9P[8] RVA in this study.

| Accession number | Strain | Genotype | Collection date | Country |
| --- | --- | --- | --- | --- |
| AB045372 | AU32 | G9 | 1986 | Japan |
| AB905466 | **BTN-88** | **G9P[8]** | **2010** | **Bhutan: Mongar** |
| DQ873674 | L169 | G9P[8] | 2004 | China：Wuhan |
| EF990708 | **B3458** | **G9P[8]** | **2003** | **Belgium** |
| EU708591 | E205 | G9P[8] | 2007 | China：Wuhan |
| EU708592 | **E192** | **G9P[8]** | **2007** | **China：Wuhan** |
| EU708593 | Z386 | G9P[8] | 2005 | China：Wuhan |
| EU708594 | **Z413** | **G9P[8]** | **2005** | **China：Wuhan** |
| EU708595 | Z434 | G9P[8] | 2006 | China：Wuhan |
| EU708596 | **L463** | **G9P[8]** | **2006** | **China：Wuhan** |
| EU708597 | SNR004 | G9P[8] | 2004 | China：Wuhan |
| EU708598 | **L720** | **G9P[8]** | **2007** | **China：Wuhan** |
| EU708599 | L865 | G9P[8] | 2007 | China：Wuhan |
| EU708600 | **E664** | **G9P[8]** | **2007** | **China：Wuhan** |
| EU708601 | L880 | G9P[8] | 2007 | China：Wuhan |
| FJ915089 | **Nov04-H429** | **G9P[6]** | **2004** | **Russia: Novosibirsk** |
| GU937089 | BJ-CR4916 | G9P[6] | 2008 | China：Beijing |
| GU937091 | **BJ-CR5317** | **G9P[8]** | **2008** | **China：Beijing** |
| HM773631 | 2009727051 | G9P[8] | 2009 | USA |
| HM773642 | **2008747307** | **G9P[8]** | **2008** | **USA** |
| HQ392002 | BE00003 | G9P[8] | 2004 | Belgium |
| HQ445973 | **Nov07-2799** | **G9P[8]** | **2007** | **Russia: Novosibirsk** |
| JN014001 | 2371WC | G9P[8] | 2008 | South Africa |
| JN605409 | **MRC-DPRU1424** | **G9P[8]** | **2009** | **Cameroon** |
| JN605420 | MRC-DPRU1723 | G9P[8] | 2009 | Zimbabwe |
| JN605431 | **MRC-DPRU4677** | **G9P[8]** | **2010** | **South Africa** |
| JN605442 | MRC-DPRU9317 | G9P[6] | 1999 | South Africa |
| JN605453 | **MRC-DPRU2427** | **G9P[8]** | **2010** | **Kenya** |
| JX185763 | ASTI23 | G9P[8] | 2007 | Italy |
| JX195068 | **AV21** | **G9P[8]** | **2010** | **Italy** |
| JX195079 | AV28 | G9P[8] | 2010 | Italy |
| JX195090 | **JES11** | **G9P[8]** | **2010** | **Italy** |
| JX411968 | H140 | G9 | 2011 | India |
| KJ412525 | **1723SR** | **G9P[8]** | **2009** | **Paraguay: Asuncion** |
| KJ412558 | 1550SR | G9P[8] | 2008 | Paraguay: Asuncion |
| KJ412591 | **1557SR** | **G9P[8]** | **2008** | **Paraguay: Asuncion** |
| KJ412636 | 1705SR | G9P[8] | 2009 | Paraguay: Asuncion |
| KJ412660 | **1604SR** | **G9P[8]** | **2008** | **Paraguay: Asuncion** |
| KJ412671 | 1720SR | G9P[8] | 2009 | Paraguay: Asuncion |
| KJ412758 | **1645SR** | **G9P[8]** | **2008** | **Paraguay: Asuncion** |
| KJ412769 | 1722SR | G9P[8] | 2009 | Paraguay: Asuncion |
| KJ559350 | **464** | **G9P[8]** | **2000** | **Paraguay: Asuncion** |
| KJ626507 | 3SR | G9P[8] | 2002 | Paraguay: Asuncion |
| KJ626518 | **1029SR** | **G9P[8]** | **2005** | **Paraguay: Asuncion** |
| KJ626540 | 533SR | G9P[8] | 2004 | Paraguay: Asuncion |
| KJ626587 | **1095SR** | **G9P[8]** | **2005** | **Paraguay: Asuncion** |
| KJ626632 | 473 | G9P[8] | 2000 | Paraguay: Asuncion |
| KJ626643 | **521SR** | **G9P[8]** | **2004** | **Paraguay: Asuncion** |
| KJ626654 | 1016SR | G9P[8] | 2005 | Paraguay: Asuncion |
| KJ626665 | **475** | **G9P[8]** | **2000** | **Paraguay: Asuncion** |
| KJ626676 | 469 | G9P[8] | 2000 | Paraguay: Asuncion |
| KJ626687 | **631SR** | **G9P[8]** | **2004** | **Paraguay: Asuncion** |
| KJ626801 | 498SR | G9P[8] | 2004 | Paraguay: Asuncion |
| KJ626812 | **1041SR** | **G9P[8]** | **2005** | **Paraguay: Asuncion** |
| KJ626823 | 468 | G9P[8] | 2000 | Paraguay: Asuncion |
| KJ626876 | **1393SR** | **G9P[8]** | **2006** | **Paraguay: Asuncion** |
| KJ626887 | 2SR | G9P[8] | 2002 | Paraguay: Asuncion |
| KJ626910 | **467** | **G9P[8]** | **2000** | **Paraguay: Asuncion** |
| KJ626921 | 615SR | G9P[8] | 2004 | Paraguay: Asuncion |
| KJ626992 | **1157A** | **G9P[4]** | **2007** | **Paraguay: Asuncion** |
| KJ627029 | 10SR | G9P[4] | 2002 | Paraguay: Asuncion |
| KJ627052 | **465** | **G9P[8]** | **2000** | **Paraguay: Asuncion** |
| KJ627094 | 1541SR | G9P[8] | 2007 | Paraguay: Asuncion |
| KJ627132 | **472** | **G9P[8]** | **2000** | **Paraguay: Asuncion** |
| KJ627143 | 1441SR | G9P[8] | 2006 | Paraguay: Asuncion |
| KJ751663 | **MRC-DPRU11070** | **G9P[6]** | **1999** | **South Africa: Ga-Rankuwa, Gauteng** |
| KJ751674 | MRC-DPRU4079-00 | G9P[6] | 2000 | South Africa: Ga-Rankuwa, Gauteng |
| KJ751707 | **MRC-DPRU384** | **G9P[6]** | **2000** | **South Africa: Ga-Rankuwa, Gauteng** |
| KJ751762 | MRC-DPRU1944 | G9P[8] | 2008 | Uganda |
| KJ751817 | **MRC-DPRU797** | **G9P[6]** | **2000** | **South Africa: Ga-Rankuwa, Gauteng** |
| KJ751965 | MRC-DPRU2126 | G9P[6] | 2003 | South Africa |
| KJ751987 | **MRC-DPRU1734** | **G9P[8]** | **2008** | **Zimbabwe** |
| KJ752111 | MRC-DPRU751 | G9P[6] | 2006 | South Africa: Ga-Rankuwa, Gauteng |
| KJ752221 | **MRC-DPRU358** | **G9P[6]** | **2000** | **South Africa: Ga-Rankuwa, Gauteng** |
| KJ752254 | MRC-DPRU9164 | G9P[6] | 1999 | South Africa: Ga-Rankuwa, Gauteng |
| KJ752300 | **MRC-DPRU3495** | **G9P[6]** | **2009** | **Zambia** |
| KJ752513 | MRC-DPRU421 | G9P[6] | 2002 | South Africa: Ga-Rankuwa, Gauteng |
| KJ752689 | **MRC-DPRU774** | **G9P[6]** | **2000** | **South Africa: Ga-Rankuwa, Gauteng** |
| KJ752797 | MRC-DPRU1841 | G9P[8] | 2009 | Zimbabwe |
| KJ752941 | **MRC-DPRU2711** | **G9P[8]** | **2008** | **South Africa: Ga-Rankuwa, Gauteng** |
| KJ753134 | MRC-DPRU2343 | G9P[8] | 2008 | South Africa: Ga-Rankuwa, Gauteng |
| KJ753315 | **MRC-DPRU3861** | **G9P[X]** | **2000** | **South Africa: Ga-Rankuwa, Gauteng** |
| KJ753373 | MRC-DPRU2943 | G9P[8] | 2008 | South Africa: Ga-Rankuwa, Gauteng |
| KJ753429 | **MRC-DPRU4595** | **G9P[8]** | **2011** | **Uganda** |
| KJ753473 | MRC-DPRU1102 | G9P[8] | 2012 | Zimbabwe |
| KJ753484 | **MRC-DPRU842** | **G9P[8]** | **2012** | **Ethiopia** |
| KJ753539 | MRC-DPRU3367 | G9P[8] | 2010 | Zimbabwe |
| KJ753710 | **MRC-DPRU3348** | **G9P[8]** | **2010** | **Zimbabwe** |
| KM008657 | KOL-20-09 | G9P[4] | 2009 | India |
| KM008658 | **KOL-29-09** | **G9P[4]** | **2009** | **India** |
| KM008659 | KOL-88-09 | G9P[8] | 2009 | India |
| KM008660 | **KOL-4-10** | **G9P[8]** | **2010** | **India** |
| KM008661 | KOL-23-10 | G9P[X] | 2010 | India |
| KM008662 | **KOL-105-10** | **G9P[8]** | **2010** | **India** |
| KM008663 | KOL-8-08 | G9P[8] | 2008 | India |
| KM008664 | **KOL-14-08** | **G9P[8]** | **2018** | **India** |
| KM008665 | KOL-42-08 | G9P[8] | 2008 | India |
| KM008666 | **KOL-52-08** | **G9P[X]** | **2008** | **India** |
| KP222830 | 21155 | G9P[8] | 2011 | Mozambique |
| KP222836 | **21162** | **G9P[8]** | **2011** | **Mozambique** |
| KP752465 | MRC-DPRU11051 | G9P[6] | 1999 | South Africa: Ga-Rankuwa, Gauteng |
| KP752521 | **MRC-DPRU5123** | **G9P[8]** | **2010** | **Senegal** |
| KP752642 | MRC-DPRU2051 | G9P[8] | 2009 | Senegal |
| KP752850 | **MRC-DPRU6825** | **G9P[6]** | **2009** | **South Africa: Ga-Rankuwa, Gauteng** |
| KP753098 | MRC-DPRU1855 | G9P[8] | 2011 | Zimbabwe |
| KP753114 | **MRC-DPRU3342** | **G9P[8]** | **2010** | **Zimbabwe** |
| KP882285 | Bang-144 | G9P[8] | 2008 | Africa |
| KP882758 | **Mali-022** | **G9P[8]** | **2008** | **Mali** |
| KP883209 | Mali-138 | G9P[8] | 2008 | Mali |
| KP941126 | **Keny-061** | **G9P[6]** | **2008** | **Kenya** |
| KT919508 | VU12-13-101 | G9P[8] | 2013 | USA: Davidson County, TN |
| KX632248 | **NSA-13-043** | **G9P[8]** | **2013** | **Uganda** |
| KX632292 | MUL-13-163 | G9P[8] | 2013 | Uganda |
| KX632303 | **MUL-12-147** | **G9P[8]** | **2012** | **Uganda** |
| KX632314 | MUL-12-093 | G9P[8] | 2012 | Uganda |
| KX632325 | **MUL-13-285** | **G9P[8]** | **2013** | **Uganda** |
| KX778597 | km15007 | G9P[8] | 2015 | China: Kunming |
| KX778598 | **km15035** | **G9P[8]** | **2015** | **China: Kunming** |
| KX778599 | km15066 | G9P[8] | 2015 | China: Kunming |
| KX778600 | **km15093** | **G9P[8]** | **2015** | **China: Kunming** |
| KX778601 | km15094 | G9P[8] | 2015 | China: Kunming |
| KX778602 | **km15095** | **G9P[8]** | **2015** | **China: Kunming** |
| KX778603 | km15097 | G9P[8] | 2015 | China: Kunming |
| KX778604 | **km15099** | **G9P[8]** | **2015** | **China: Kunming** |
| KX778605 | km15100 | G9P[8] | 2015 | China: Kunming |
| KX778606 | **km15105** | **G9P[8]** | **2015** | **China: Kunming** |
| KX778607 | km15118 | G9P[8] | 2016 | China: Kunming |
| KX778608 | **km15119** | **G9P[8]** | **2016** | **China: Kunming** |
| LC105522 | UR14-25 | G9P[8] | 2014 | Japan |
| LC172287 | **NT062** | **G9P[8]** | **2013** | **Japan** |
| LC172364 | OT007 | G9P[8] | 2013 | Japan |
| LC172396 | **SP010** | **G9P[8]** | **2013** | **Japan** |
| LC172422 | YM017 | G9P[8] | 2013 | Japan |
| LC172445 | **KN160** | **G9P[8]** | **2014** | **Japan** |
| LC172449 | KN164 | G9P[8] | 2014 | Japan |
| LC172451 | **YM076** | **G9P[8]** | **2014** | **Japan** |
| LC172452 | YM077 | G9P[8] | 2014 | Japan |
| LC172457 | **YR173** | **G9P[8]** | **2014** | **Japan** |
| LC172458 | YR175 | G9P[8] | 2014 | Japan |
| LC228386 | **CH1023** | **G9P[8]** | **2016** | **Japan** |
| LC228397 | IS1080 | G9P[8] | 2016 | Japan |
| LC228408 | **MI1128** | **G9P[8]** | **2016** | **Japan** |
| LC311228 | To16-02 | G9P[8] | 2016 | Japan |
| LC514475 | **DBM2017-016** | **G9P[8]** | **2017** | **Thailand:Bangkok** |
| LC568862 | MU16-01 | G9P[8] | 2016 | Japan |
| LC568863 | **MU16-03** | **G9P[8]** | **2016** | **Japan** |
| LC568864 | NS16-01 | G9P[8] | 2016 | Japan |
| LC568872 | **TA16-02** | **G9P[8]** | **2016** | **Japan** |
| LC568873 | To16-09 | G9P[8] | 2016 | Japan |
| LC568882 | **UR16-01** | **G9P[8]** | **2016** | **Japan** |
| LC568887 | HK16-02 | G9P[8] | 2016 | Japan |
| LC568903 | **Ho16-02** | **G9P[8]** | **2016** | **Japan** |
| LC568905 | IW16-01 | G9P[8] | 2016 | Japan |
| LC568909 | **IW16-12** | **G9P[8]** | **2016** | **Japan** |
| LC568910 | RU16-01 | G9P[8] | 2016 | Japan |
| LC568919 | **SU16-03** | **G9P[8]** | **2016** | **Japan** |
| LC569526 | RU17-08 | G9P[8] | 2017 | Japan |
| LC569529 | **MU17-03** | **G9P[8]** | **2017** | **Japan** |
| MG652303 | 3000503696 | G9P[8] | 2014 | Dominican Republic |
| MG652304 | **3000503700** | **G9P[8]** | **2014** | **Dominican Republic** |
| MH109856 | PAK56 | G9P[8] | 2015 | Pakistan |
| MH291284 | **3471** | **G9P[8]** | **2016** | **Kenya** |
| MH291285 | 3468 | G9P[8] | 2016 | Kenya |
| MH712920 | **IDH_6311** | **G9** | **2014** | **India** |
| MK560910 | BCH_5567 | G9 | 2015 | India |
| MK560919 | **BCH_4166** | **G9** | **2014** | **India** |
| MK560920 | IDH_9733 | G9 | 2017 | India |
| MN066759 | **CMC_00048** | **G9P[8]** | **2013** | **India: Vellore** |
| MN066996 | CMC_00005 | G9P[X] | 2011 | India: Vellore |
| MN067177 | **CMC_00006** | **G10P[X]** | **2011** | **India: Vellore** |
| MN067203 | CMC_00007 | G9P[8] | 2011 | India: Vellore |
| MN106113 | **E6356** | **G9P[8]** | **2019** | **China：Wuhan** |
| MN106114 | E6398 | G9P[8] | 2019 | China：Wuhan |
| MN106115 | **L2448** | **G9P[8]** | **2019** | **China：Wuhan** |
| MN106116 | Z2768 | G9P[8] | 2019 | China：Wuhan |
| MN194490 | **KLF0667** | **G9P[8]** | **2013** | **Kenya** |
| MN478547 | 3000014466 | G9P[8] | 2014 | USA: Seattle |
| MN478548 | **3000053718** | **G9P[8]** | **2015** | **USA: Vanderbilt** |
| MN478587 | 3000357125 | G9P[8] | 2016 | USA: Oakland |
| MN478592 | **3000368549** | **G9P[8]** | **2015** | **USA: Rochester** |
| MN478593 | 3000368927 | G9P[8] | 2015 | USA: Rochester |
| MN478606 | **3000380486** | **G9P[8]** | **2015** | **USA: Seattle** |
| MN478616 | 3000449274 | G9P[8] | 2015 | USA: Seattle |
| MN478620 | **3000490361** | **G9P[8]** | **2016** | **USA: Oakland** |
| MN478621 | 3000490366 | G9P[8] | 2016 | USA: Oakland |
| MN478622 | **3000524336** | **G9P[8]** | **2016** | **USA: Rochester** |
| MN478627 | 3000526249 | G9P[8] | 2016 | USA: Vanderbilt |
| MN529645 | **JZ1812** | **G9P[8]** | **2018** | **China：Jinzhou** |
| MN529646 | JZ1811 | G9P[8] | 2018 | China：Jinzhou |
| MN529647 | **JZ1903** | **G9P[8]** | **2019** | **China：Jinzhou** |
| MN551926 | NS16-C7 | G9P[8] | 2016 | Russia: Novosibirsk |
| MN552032 | **NS16-A274** | **G9P[8]** | **2016** | **Russia: Novosibirsk** |
| MN552061 | NS16-A750 | G9P[8] | 2016 | Russia: Novosibirsk |
| MN552061 | **NS16-A750** | **G9P[8]** | **2016** | **Russia: Novosibirsk** |
| MN552077 | NS17-A866 | G9P[8] | 2017 | Russia: Novosibirsk |
| MN552087 | **NS17-A895** | **G9P[8]** | **2017** | **Russia: Novosibirsk** |
| MN552103 | NS17-A959 | G9P[8] | 2017 | Russia: Novosibirsk |
| MN577086 | **NS17-A1264** | **G9P[8]** | **2017** | **Russia: Novosibirsk** |
| MN577087 | NS18-A1411 | G9P[8] | 2018 | Russia: Novosibirsk |
| MT005292 | **H186** | **G9P[4]** | **2018** | **Czech Republic** |
| MT005303 | H187 | G9P[4] | 2018 | Czech Republic |
| MT107163 | **JZ1810** | **G9P[8]** | **2018** | **China：Jinzhou** |
| MT107164 | JZ1901 | G9P[8] | 2019 | China：Jinzhou |
| MT107165 | **JZ1911** | **G9P[8]** | **2019** | **China：Jinzhou** |
| MZ093873 | KLF0561 | G9P[8] | 2012 | Kenya |
| MZ095086 | **KLF0701** | **G9P[8]** | **2014** | **Kenya** |
| MZ095348 | KLF0755 | G9P[8] | 2015 | Kenya |
| MZ096909 | **KLF1010** | **G9P[8]** | **2014** | **Kenya** |

**Table S3** The 134 VP4 strains used for evolutionary analyses in the MCC tree of G9P[8] RVA in this study.

| Accession number | Strain | Genotype | Collection date | Country |
| --- | --- | --- | --- | --- |
| AB975469 | NT009 | G3P[8] | 2012 | Japan |
| AB975477 | **NT020** | **G1P[8]** | **2012** | **Japan** |
| AB975486 | KN008 | G9P[8] | 2012 | Japan |
| FJ623201 | **951454** | **G1P[8]** | **1995** | **India** |
| FJ947873 | DC2262 | G3P[8] | 1976 | USA |
| GQ453422 | **Nov09-D10** | **G9P[8]** | **2009** | **Russia: Novosibirsk** |
| GQ996805 | CU328-NR | G9P[8] | 2008 | Thailand |
| HM773615 | **2009727047** | **G9P[8]** | **2009** | **USA** |
| HM773626 | 2009727051 | G9P[8] | 2009 | USA |
| HM773659 | **2008747369** | **G3P[8]** | **2008** | **USA** |
| HM773725 | 2009726997 | G3P[8] | 2009 | USA |
| HM773736 | **2008747322** | **G3P[8]** | **2008** | **USA** |
| HM773813 | 2007744509 | G1P[8] | 2007 | USA |
| HM773824 | **2007744270** | **G1P[8]** | **2007** | **USA** |
| HM773835 | 2007719945 | G1P[8] | 2007 | USA |
| HM773846 | **2007719907** | **G1P[8]** | **2007** | **USA** |
| HQ392409 | BE00045 | G1P[8] | 2009 | Belgium: Flanders |
| HQ738573 | **Nov10-N205** | **G4P[8]** | **2010** | **Russia: Novosibirsk** |
| HQ881574 | NIV-07523 | G1P[8] | 2007 | India |
| JF813100 | **E1545** | **G9P[8]** | **2009** | **China** |
| JN849149 | BE1280 | G1P[8] | 2009 | Belgium |
| JN849151 | **BE1520** | **G1P[8]** | **2009** | **Belgium** |
| JQ069460 | RT005-07 | G1P[8] | 2007 | Canada |
| JQ069622 | **RT004-07** | **G3P[8]** | **2007** | **Canada** |
| JQ069669 | RT131-07 | G3P[8] | 2008 | Canada |
| JQ069685 | **RT010-09** | **G3P[8]** | **2009** | **Canada** |
| JQ248941 | Nov09-D278 | G1P[8] | 2009 | Russia: Novosibirsk |
| JX027887 | **CK00089** | **G1P[8]** | **2009** | **Australia: Melbourne, Victoria** |
| JX027933 | CK00095 | G1P[8] | 2010 | Australia: Melbourne, Victoria |
| KC442955 | **VU08-09-22** | **G2P[8]** | **2008** | **USA: Vanderbilt** |
| KF371744 | E093 | G3P[8] | 2007 | China: Wuhan |
| KF371832 | **E2421** | **G3P[8]** | **2010** | **China: Wuhan** |
| KF371854 | E2432 | G3P[8] | 2010 | China: Wuhan |
| KF371876 | **E2835** | **G3P[8]** | **2011** | **China: Wuhan** |
| KF371887 | E3239 | G3P[8] | 2012 | China: Wuhan |
| KF371984 | **R1604** | **G3P[8]** | **2011** | **China: Wuhan** |
| KF372006 | Z1557 | G3P[8] | 2011 | China: Wuhan |
| KF372017 | **Z1602** | **G3P[8]** | **2012** | **China: Wuhan** |
| KF648954 | Nov09-D386 | G1P[8] | 2009 | Russia: Novosibirsk |
| KJ583177 | **Arg7338** | **G3P[8]** | **2009** | **Argentina** |
| KJ752230 | MRC-DPRU1840-07 | G1P[8] | 2007 | South Africa: Ga-Rankuwa, Gauteng |
| KJ753161 | **MRC-DPRU2132** | **G1P[8]** | **2005** | **South Africa: Ga-Rankuwa, Gauteng** |
| KJ753471 | MRC-DPRU1102 | G9P[8] | 2012 | Zimbabwe |
| KM116029 | **2013774164** | **G12P[8]** | **2013** | **USA** |
| KM116040 | 2013774165 | G12P[8] | 2013 | USA |
| KM116051 | **2013774166** | **G12P[8]** | **2013** | **USA** |
| KP752519 | MRC-DPRU5123 | G9P[8] | 2010 | Togo |
| KP902535 | **MW670** | **G4P[8]** | **1999** | **Malawi** |
| KP902538 | R6293 | G9P[8] | 2010 | Israel |
| KP902539 | **R5808** | **G9P[8]** | **2009** | **Israel** |
| KP902549 | MRC-DPRU2144 | G9P[8] | 2003 | South Africa |
| KT920621 | **CNMC114** | **G1P[8]** | **2011** | **USA: Washington, DC** |
| KT920621 | CNMC114 | G1P[8] | 2011 | USA: Washington, DC |
| KT920687 | **CNMC125** | **G1P[8]** | **2011** | **USA: Washington, DC** |
| KT988164 | PR1470 | G3P[8] | 2009 | Italy |
| KT988252 | **PR599** | **G3P[8]** | **2013** | **Italy** |
| KX646599 | RV1013 | G9P[8] | 2010 | India |
| KX778582 | **km15105** | **G9P[8]** | **2015** | **China: Kunming** |
| LC066159 | SP071 | G1P[8] | 2012 | Viet Nam |
| LC066195 | **SP118** | **G1P[8]** | **2013** | **Viet Nam** |
| LC105045 | HK14-9 | G1P[8] | 2014 | Japan:Hokkaido, Sapporo |
| LC172581 | **OT045** | **G9P[8]** | **2013** | **Japan** |
| LC172581 | OT045 | G9P[8] | 2013 | Japan |
| LC172616 | **YM026** | **G9P[8]** | **2013** | **Japan** |
| LC172633 | KN160 | G9P[8] | 2014 | Japan |
| LC172639 | **YM076** | **G9P[8]** | **2014** | **Japan** |
| LC228384 | CH1023 | G9P[8] | 2016 | Japan |
| LC260224 | **SOEP075** | **G3P[8]** | **2016** | **Indonesia** |
| LC477380 | Tokyo16-3571 | G9P[8] | 2017 | Japan:Tokyo |
| LC477384 | **Tokyo16-4754** | **G9P[8]** | **2017** | **Japan:Tokyo** |
| LC477385 | Tokyo17-08 | G3P[8] | 2017 | Japan:Tokyo |
| LC477397 | **Tokyo18-27** | **G9P[8]** | **2018** | **Japan:Tokyo** |
| LC477398 | Tokyo18-30 | G9P[8] | 2018 | Japan:Tokyo |
| LC477401 | **Tokyo18-37** | **G9P[8]** | **2018** | **Japan:Tokyo** |
| LC477402 | Tokyo18-38 | G9P[8] | 2018 | Japan:Tokyo |
| LC477407 | **Tokyo18-43** | **G9P[8]** | **2018** | **Japan:Tokyo** |
| LC477409 | Tokyo18-50 | G9P[8] | 2018 | Japan:Tokyo |
| LC491485 | **RVN16.1164** | **G9P[8]** | **2016** | **Viet Nam** |
| LC491496 | RVN16.1467 | G9P[8] | 2016 | Viet Nam |
| LC491539 | **RVN18.0181** | **G9P[8]** | **2018** | **Viet Nam** |
| LC514550 | DBM2018-111 | G9P[8] | 2018 | Thailand:Bangkok |
| LC546072 | **RVA-U7** | **G9P[8]** | **2017** | **Japan** |
| LC568931 | To16-02 | G9P[8] | 2016 | Japan:Hokkaido |
| LC568965 | **IW16-01** | **G9P[8]** | **2016** | **Japan:Hokkaido** |
| MG816515 | SC9 | P[8] | 2014 | China: Chengdu |
| MG816516 | **SC10** | **P[8]** | **2013** | **China: Chengdu** |
| MG816517 | SC11 | P[8] | 2013 | China: Chengdu |
| MH182439 | **PAK56** | **G9P[8]** | **2015** | **Pakistan** |
| MK601619 | JS2012 | G9P[8] | 2012 | China: Jiangsu |
| MN106118 | **E5365** | **G1P[8]** | **2017** | **China: Wuhan** |
| MN106119 | E5867 | G3P[8] | 2018 | China: Wuhan |
| MN106120 | **E6356** | **G9P[8]** | **2019** | **China: Wuhan** |
| MN106121 | E6398 | G9P[8] | 2019 | China: Wuhan |
| MN106122 | **L2448** | **G9P[8]** | **2019** | **China: Wuhan** |
| MN106123 | Z2768 | G9P[8] | 2019 | China: Wuhan |
| MN295464 | **EcWt-O** | **G1P[8]** | **2013** | **Colombia** |
| MN478704 | 3000014466 | G9P[8] | 2014 | USA: Seattle |
| MN478744 | **3000357125** | **G9P[8]** | **2016** | **USA: Seattle** |
| MN478750 | 3000368927 | G9P[8] | 2015 | USA: Seattle |
| MN478763 | **3000380486** | **G9P[8]** | **2015** | **USA: Seattle** |
| MN552140 | NS17-A1215 | G1P[8] | 2017 | Russia: Novosibirsk |
| MT107168 | **JZ1911** | **G9P[8]** | **2019** | **China** |
| MT107169 | JZ2001 | G9P[8] | 2020 | China |
| MT854974 | **MRC-DPRU3433** | **G1P[8]** | **2006** | **South Africa** |
| MW280953 | PA95 | G3P[8] | 2013 | Italy |
| MZ165472 | **HB-19** | **G9P[8]** | **2019** | **China** |
| MZ165473 | HB-20 | G9P[8] | 2019 | China |
| MZ165475 | **HB-27** | **G9P[8]** | **2019** | **China** |
| MZ165478 | HB-45 | G9P[8] | 2019 | China |
| MZ165480 | **HB-109** | **G9P[8]** | **2019** | **China** |
| OM037828 | FJ16351342 | G9P[8] | 2016 | China: Fuzhou |
| OM037839 | **GX16451148** | **G3P[8]** | **2016** | **China: Luocheng** |
| OM037850 | GX16451181 | G3P[8] | 2016 | China: Luocheng |
| OM037861 | **GX16451194** | **G3P[8]** | **2016** | **China: Luocheng** |
| OM037872 | HEB16231045 | G3P[8] | 2016 | China: Harbin |
| OM037883 | **HEB16231054** | **G3P[8]** | **2016** | **China: Harbin** |
| OM037894 | NM16151098 | G3P[8] | 2016 | China: Huhhot |
| OM037905 | **NM16151108** | **G3P[8]** | **2016** | **China: Huhhot** |
| OM037916 | SC16511014 | G3P[8] | 2016 | China: Chengdu |
| OM037927 | **SC16511020** | **G3P[8]** | **2016** | **China: Chengdu** |
| OM037938 | SC16511029 | G3P[8] | 2016 | China: Chengdu |
| OM037949 | **SC16511036** | **G3P[8]** | **2016** | **China: Chengdu** |
| OM037960 | SC16511041 | G3P[8] | 2016 | China: Chengdu |
| OM037971 | **SC16511047** | **G3P[8]** | **2016** | **China: Chengdu** |
| OM037982 | SC16511058 | G3P[8] | 2016 | China: Chengdu |
| OM037993 | **SC16511067** | **G3P[8]** | **2016** | **China: Chengdu** |
| OM038004 | SC18511082 | G3P[8] | 2018 | China: Chengdu |
| OM038015 | **SC18511069** | **G3P[8]** | **2018** | **China: Chengdu** |
| OM038026 | SC18511047 | G3P[8] | 2018 | China: Chengdu |
| OM038037 | **SC18511027** | **G3P[8]** | **2018** | **China: Chengdu** |
| OM038048 | JL18221081 | G3P[8] | 2018 | China: Changchun |
| OM038059 | **GD18442042** | **G3P[8]** | **2018** | **China: Guangzhou** |
| OM038070 | GD18442014 | G3P[8] | 2018 | China: Guangzhou |
| OM038081 | **GD18442005** | **G3P[8]** | **2018** | **China: Guangzhou** |

**Table S4** The 96 NSP4-E1 strains used for evolutionary analyses in the MCC tree of G9P[8] RVA in this study.

| Accession number | Strain | Genotype | Collection date | Country |
| --- | --- | --- | --- | --- |
| JN258797 | **BE00055** | **1999** | **G1P[8]** | **Belgium: Flanders** |
| JQ087432 | Y128 | 2004 | G1P[8] | China |
| JX027750 | **CK00074** | **2007** | **G1P[8]** | **Australia: Melbourne, Victoria** |
| KC579507 | DC3669 | 1989 | G1P[8] | USA: Washington, DC |
| KC579642 | **DC4315** | **1988** | **G1P[8]** | **USA: Washington, DC** |
| KC579643 | DC4315 | 1988 | G1P[8] | USA: Washington, DC |
| KC579663 | **DC5385** | **1991** | **G1P[8]** | **USA: Washington, DC** |
| KC579700 | DC3723 | 1989 | G1P[8] | USA: Washington, DC |
| KC579790 | **DC3721** | **1989** | **G1P[8]** | **USA: Washington, DC** |
| KC579889 | DC1228 | 1980 | G1P[8] | USA: Washington, DC |
| KC579927 | **DC4312** | **1988** | **G1P[8]** | **USA: Washington, DC** |
| KC580036 | DC3779 | 1989 | G1P[8] | USA: Washington, DC |
| KC580037 | **DC3779** | **1989** | **G1P[8]** | **USA: Washington, DC** |
| KC580320 | DC3855 | 1989 | G1P[8] | USA: Washington, DC |
| KC580321 | **DC3855** | **1989** | **G1P[8]** | **USA: Washington, DC** |
| KC580432 | DC1292 | 1980 | G1P[8] | USA: Washington, DC |
| KF371849 | **E2432** | **2010** | **G3P[8]** | **China: Wuhan** |
| KF371882 | E3239 | 2012 | G3P[8] | China: Wuhan |
| KF371882 | **E3239** | **2012** | **G3P[8]** | **China: Wuhan** |
| KF371924 | R1267 | 2006 | G3P[8] | China: Wuhan |
| KF371968 | **R303** | **2004** | **G3P[8]** | **China: Wuhan** |
| KF372001 | Z1557 | 2011 | G3P[8] | China: Wuhan |
| KF372012 | **Z1602** | **2012** | **G3P[8]** | **China: Wuhan** |
| KF372012 | Z1602 | 2012 | G3P[8] | China: Wuhan |
| KF726053 | **E2484** | **2011** | **G4P[8]** | **China: Wuhan** |
| KJ094908 | LL10131400 | 2010 | G9P[8] | China: Hebei Lulong |
| KJ627148 | **PRY/55** | **1998** | **G1P[8]** | **Paraguay: Asuncion** |
| KT223437 | BE00059 | 1999 | G1P[8] | Belgium |
| KT695003 | **DC4455** | **1988** | **G1P[8]** | **USA: Washington, DC** |
| KT695058 | DC3695 | 1989 | G1P[8] | USA: Washington, DC |
| KT695113 | **DC5685** | **1991** | **G1P[8]** | **USA: Washington, DC** |
| LC105020 | HK14-5 | 2014 | G1P[8] | Japan:Hokkaido, Sapporo |
| LC105064 | **HK14-10** | **2014** | **G1P[8]** | **Japan:Hokkaido, Sapporo** |
| LC105409 | To14-38 | 2014 | G1P[8] | Japan:Hokkaido, Tomakomai |
| LC105487 | **UR14-20** | **2014** | **G9P[8]** | **Japan:Hokkaido, Urakawa** |
| LC105531 | UR14-26 | 2014 | G9P[8] | Japan:Hokkaido, Urakawa |
| LC173963 | **NT036** | **2013** | **G1P[8]** | **Japan** |
| LC173981 | NT064 | 2013 | G1P[8] | Japan |
| LC174100 | **SP024** | **2013** | **G1P[8]** | **Japan** |
| LC174120 | YM026 | 2013 | G9P[8] | Japan |
| LC174125 | **YM043** | **2013** | **G9P[8]** | **Japan** |
| LC174127 | YM048 | 2013 | G9P[8] | Japan |
| LC174128 | **YM051** | **2013** | **G9P[8]** | **Japan** |
| LC174137 | KN160 | 2014 | G9P[8] | Japan |
| LC174144 | **YM077** | **2014** | **G9P[8]** | **Japan** |
| LC368105 | 5N0109 | 2005 | G12P[6] | Nepal |
| LC372867 | **5N0031** | **2005** | **G12P[6]** | **Nepal** |
| LC477624 | Tokyo16-4754 | 2017 | G9P[8] | Japan:Tokyo |
| LC477625 | **Tokyo17-08** | **2017** | **G3P[8]** | **Japan:Tokyo** |
| LC477649 | Tokyo18-50 | 2018 | G9P[8] | Japan:Tokyo |
| LC491493 | **RVN16.1164** | **2016** | **G9P[8]** | **Viet Nam** |
| LC491504 | RVN16.1467 | 2016 | G9P[8] | Viet Nam |
| LC569401 | **MU16-03** | **2016** | **G9P[8]** | **Japan:Hokkaido prefecture, Muroran city** |
| LC569402 | NS16-01 | 2016 | G9P[8] | Japan:Hokkaido prefecture, Sapporo city |
| LC569403 | **NS16-09** | **2016** | **G9P[8]** | **Japan:Hokkaido prefecture, Sapporo city** |
| LC569408 | NS16-22 | 2016 | G9P[8] | Japan:Hokkaido prefecture, Sapporo city |
| LC569411 | **To16-02** | **2016** | **G9P[8]** | **Japan:Hokkaido prefecture, Tomakomai city** |
| LC569416 | To16-21 | 2016 | G9P[8] | Japan:Hokkaido prefecture, Tomakomai city |
| LC569420 | **To16-34** | **2016** | **G9P[8]** | **Japan:Hokkaido prefecture, Tomakomai city** |
| LC569423 | UR16-02 | 2016 | G9P[8] | Japan:Hokkaido prefecture, Urakawa city |
| LC569425 | **UR16-06** | **2016** | **G9P[8]** | **Japan:Hokkaido prefecture, Urakawa city** |
| LC569427 | HK16-02 | 2016 | G9P[8] | Japan:Hokkaido prefecture, Sapporo city |
| LC569430 | **HK16-17** | **2016** | **G9P[8]** | **Japan:Hokkaido prefecture, Sapporo city** |
| LC569434 | HK16-50 | 2016 | G9P[8] | Japan:Hokkaido prefecture, Sapporo city |
| LC569436 | **HK16-52** | **2016** | **G9P[8]** | **Japan:Hokkaido prefecture, Sapporo city** |
| LC569437 | HK16-54 | 2016 | G9P[8] | Japan:Hokkaido prefecture, Sapporo city |
| LC569441 | **HK16-69** | **2016** | **G9P[8]** | **Japan:Hokkaido prefecture, Sapporo city** |
| LC569443 | Ho16-02 | 2016 | G9P[8] | Japan:Hokkaido prefecture, Sapporo city |
| LC569446 | **IW16-03** | **2016** | **G9P[8]** | **Japan:Hokkaido prefecture, Iwamizawa city** |
| LC569451 | RU16-02 | 2016 | G9P[8] | Japan:Hokkaido prefecture, Rumoi city |
| LC569454 | **RU16-17** | **2016** | **G9P[8]** | **Japan:Hokkaido prefecture, Rumoi city** |
| LC569456 | RU16-24 | 2016 | G9P[8] | Japan:Hokkaido prefecture, Rumoi city |
| LC569456 | **RU16-24** | **2016** | **G9P[8]** | **Japan:Hokkaido prefecture, Rumoi city** |
| LC569459 | SU16-03 | 2016 | G9P[8] | Japan:Hokkaido prefecture, Iwamizawa city |
| LC569620 | **To17-01** | **2017** | **G1P[8]** | **Japan:Hokkaido prefecture, Tomakomai city** |
| LC569623 | RU17-08 | 2017 | G9P[8] | Japan:Hokkaido prefecture, Rumoi city |
| MF580889 | **JS2010** | **2010** | **G9P[8]** | **China** |
| MF580895 | JS2016 | 2016 | G9P[8] | China |
| MH094760 | **20130113** | **2013** | **G1P[4]** | **China** |
| MN106175 | E5867 | 2018 | G3P[8] | China: Wuhan |
| MN106176 | **E6356** | **2019** | **G9P[8]** | **China: Wuhan** |
| MT292025 | COD00155 | 1991 | G1P[8] | Brazil |
| MZ165413 | **HB-19** | **2019** | **G9P[8]** | **China** |
| MZ165415 | HB-20 | 2019 | G9P[8] | China |
| MZ165419 | **HB-7** | **2019** | **G9P[8]** | **China** |
| OM037834 | FJ16351342 | 2016 | G3P[8] | China: Fujian |
| OM037845 | **GX16451148** | **2016** | **G3P[8]** | **China: Guangxi** |
| OM037856 | GX16451181 | 2016 | G3P[8] | China: Guangxi |
| OM037867 | **GX16451194** | **2016** | **G3P[8]** | **China: Guangxi** |
| OM037878 | HEB16231045 | 2016 | G3P[8] | China: Heilongjiang |
| OM037889 | **HEB16231054** | **2016** | **G3P[8]** | **China: Heilongjiang** |
| OM037900 | NM16151098 | 2016 | G3P[8] | China: Inner Mongolia |
| OM037911 | **NM16151108** | **2016** | **G3P[8]** | **China: Inner Mongolia** |
| OM037922 | SC16511014 | 2016 | G3P[8] | China: Sichuan |
| OM037933 | **SC16511020** | **2016** | **G3P[8]** | **China: Sichuan** |
| OM037944 | SC16511029 | 2016 | G3P[8] | China: Sichuan |

**Table S5** The 100 NSP4-E2 strains used for evolutionary analyses in the MCC tree of G9P[8] RVA in this study.

| Accession number | Strain | Genotype | Collection date | Country |
| --- | --- | --- | --- | --- |
| GQ240626 | mani-265 | G10P[6] | 2007 | India: Manipur |
| GQ428139 | IRL | G10P[11] | 2007 | Ireland |
| JQ043301 | CMH028 | G2P[4] | 2007 | Thailand |
| JQ043301 | CMH028/07 | G2P[4] | 2007 | Thailand |
| JQ837886 | BL-5210 | G2P[4] | 2006 | Indonesia |
| JQ837886 | BL-5210 | G2P[4] | 2006 | Indonesia |
| JX965154 | WAPC703 | G2P[4] | 2010 | Australia |
| JX965154 | WAPC703 | G2P[4] | 2010 | Australia |
| KC178745 | PA130 | G2P[4] | 2010 | Italy |
| KC178750 | PA108 | G2P[4] | 2007 | Italy |
| KC178751 | PA17 | G2P[4] | 2008 | Italy |
| KC178751 | PA17 | G2P[4] | 2008 | Italy |
| KC443782 | CK20051 | G2P[4] | 2010 | Australia: Melbourne, Victoria |
| KC571500 | SA066 | G2P[4] | 2010 | Australia |
| KF690134 | RCH272 | G3P[14] | 2012 | Australia |
| KP007157 | TGO12-003 | G2P[4] | 2012 | Philippines |
| KP007168 | TGO12-004 | G1P[8] | 2012 | Philippines |
| KP007189 | TGO12-012 | G1P[8] | 2012 | Philippines |
| KP013444 | Hv2258625 | G9P[4] | 2010 | Denmark |
| KP013444 | Hv2258625 | G9P[4] | 2010 | Denmark |
| KP752658 | MRC-DPRU295 | G2P[4] | 2012 | Mauritius |
| KP753233 | MRC-DPRU296 | G2P[4] | 2012 | Mauritius |
| KU248403 | J266 | G2P[4] | 2010 | Bangladesh |
| KU248425 | J263 | G2P[4] | 2010 | Bangladesh |
| KU248437 | J331 | G2P[4] | 2010 | Bangladesh |
| KU356616 | M313 | G2P[4] | 2013 | Bangladesh |
| KU356627 | M312 | G2P[4] | 2013 | Bangladesh |
| KU361023 | QUI-36-F1 | G2P[4] | 2008 | Brazil: Para, Ananindeua |
| KU361024 | QUI-59-F1 | G2P[4] | 2008 | Brazil: Para, Ananindeua |
| KU361025 | QUI-60-F1 | G2P[4] | 2008 | Brazil: Para, Ananindeua |
| KX347174 | SS96085815 | G2P[4] | 2015 | Spain: Gipuzkoa, Basque Country |
| KX347174 | SS96085815 | G2P[4] | 2015 | Spain: Gipuzkoa, Basque Country |
| KX638734 | RV0904 | G2P[4] | 2009 | India |
| KX638741 | RV1206 | G2P[4] | 2012 | India |
| KX638741 | RV1206 | G2P[4] | 2012 | India |
| KY497560 | PAK93 | G1P[6] | 2010 | Pakistan |
| KY497560 | PAK93 | G1P[6] | 2010 | Pakistan |
| LC066156 | SP026 | G1P[8] | 2012 | Viet Nam |
| LC105139 | MU14-13 | G2P[4] | 2014 | Japan:Hokkaido, Muroran |
| LC105161 | MU14-15 | G2P[4] | 2014 | Japan:Hokkaido, Muroran |
| LC105172 | MU14-16 | G2P[4] | 2014 | Japan:Hokkaido, Muroran |
| LC105172 | MU14-16 | G2P[4] | 2014 | Japan:Hokkaido, Muroran |
| LC105234 | MU14-22 | G2P[4] | 2014 | Japan:Hokkaido, Muroran |
| LC105290 | To14-24 | G2P[4] | 2014 | Japan:Hokkaido, Tomakomai |
| LC105290 | To14-24 | G2P[4] | 2014 | Japan:Hokkaido, Tomakomai |
| LC174090 | SP012 | G2P[4] | 2013 | Japan |
| LC174138 | KN161 | G2P[4] | 2014 | Japan |
| LC174139 | KN162 | G2P[4] | 2014 | Japan |
| LC174146 | YM086 | G2P[4] | 2014 | Japan |
| LC174148 | YM090 | G2P[4] | 2014 | Japan |
| LC260310 | SOEP033 | G3P[8] | 2015 | Indonesia |
| LC469567 | STM182 | G3P[6] | 2016 | Indonesia: East Java |
| LC469576 | D37 | G1P[8] | 2013 | Indonesia: East Java |
| LC477622 | Tokyo16-4415 | G2P[4] | 2017 | Japan:Tokyo |
| LC477627 | Tokyo17-10 | G2P[4] | 2017 | Japan:Tokyo |
| LC477629 | Tokyo17-15 | G2P[4] | 2017 | Japan:Tokyo |
| LC477638 | Tokyo18-30 | G9P[8] | 2018 | Japan:Tokyo |
| LC477642 | Tokyo18-38 | G9P[8] | 2018 | Japan:Tokyo |
| LC477643 | Tokyo18-39 | G9P[8] | 2018 | Japan:Tokyo |
| LC477643 | Tokyo18-39 | G9P[8] | 2018 | Japan:Tokyo |
| LC477647 | Tokyo18-43 | G9P[8] | 2018 | Japan:Tokyo |
| LC514501 | DBM2018-291 | G9P[8] | 2018 | Thailand:Bangkok |
| LC514501 | DBM2018-291 | G9P[8] | 2018 | Thailand:Bangkok |
| LC514512 | DBM2017-003 | G2P[4] | 2017 | Thailand:Bangkok |
| LC514512 | DBM2017-003 | G2P[4] | 2017 | Thailand:Bangkok |
| LC514523 | DBM2017-015 | G2P[4] | 2017 | Thailand:Bangkok |
| LC514534 | DBM2018-105 | G2P[4] | 2018 | Thailand:Bangkok |
| LC514534 | DBM2018-105 | G2P[4] | 2018 | Thailand:Bangkok |
| LC569433 | HK16-47 | G2P[4] | 2016 | Japan:Hokkaido prefecture, Sapporo city |
| LC569629 | HK17-18 | G2P[4] | 2017 | Japan:Hokkaido prefecture, Sapporo city |
| LC668558 | MI1161 | G2P[4] | 2018 | Japan:Mie |
| MF469133 | SSCRTV_00011 | G2P[4] | 2013 | USA: Memphis, TN |
| MF469133 | SSCRTV_00011 | G2P[4] | 2013 | USA: Memphis, TN |
| MG573368 | IAL-R3123 | G1P[8] | 2013 | Brazil |
| MH182463 | PAK419 | G3P[4] | 2016 | Pakistan |
| MH182466 | PAK663 | G3P[4] | 2016 | Pakistan |
| MH520739 | IAL-R530 | G3P[8] | 2016 | Brazil |
| MN066887 | CMC_00043 | G2P[X] | 2012 | India: Vellore |
| MN066905 | CMC_00017 | GXP[X] | 2011 | India: Vellore |
| MN067026 | CMC_00019 | G2P[X] | 2012 | India: Vellore |
| MN067089 | CMC_00016 | G2P[X] | 2011 | India: Vellore |
| MN067141 | CMC_00027 | G2P[8] | 2012 | India: Vellore |
| MN067181 | CMC_00018 | G2P[X] | 2012 | India: Vellore |
| MN106177 | E6398 | G9P[8] | 2019 | China: Wuhan |
| MN106178 | L2448 | G9P[8] | 2019 | China: Wuhan |
| MN106179 | Z2768 | G9P[8] | 2019 | China: Wuhan |
| MN106179 | Z2768 | G9P[8] | 2019 | China: Wuhan |
| MN206138 | 557 | G2P[4] | 2017 | Russia |
| MN529657 | JZ1812 | G9P[8] | 2019 | China: Jinzhou |
| MN529658 | JZ1811 | G9P[8] | 2019 | China: Jinzhou |
| MN529659 | JZ1903 | G9P[8] | 2019 | China: Jinzhou |
| MW280894 | PA213 | G3P[8] | 2017 | Italy |
| MW384009 | Fuzhou18-113 | G9P[8] | 2018 | China: Fuzhou |
| MW384108 | Fuzhou18-152 | G9P[8] | 2018 | China: Fuzhou |
| MW384185 | Fuzhou18-96 | G9P[8] | 2018 | China: Fuzhou |
| MW384328 | Fuzhou19-38 | G9P[8] | 2019 | China: Fuzhou |
| MZ066376 | 3001607823 | G2P[4] | 2018 | Benin |
| MZ165407 | HB-1 | GXP[X] | 2019 | China |
| MZ165410 | HB-45 | G9P[8] | 2019 | China |
| OK244037 | B5383 | G3P[4] | 2018 | Thailand |
